# Consensus molecular subtypes of primary colon tumors and their hepatic metastases

**DOI:** 10.2144/fsoa-2021-0021

**Published:** 2021-05-28

**Authors:** Yee Chen Lau, Sebastian Schmeier, Frank Frizelle, Rachel Purcell

**Affiliations:** 1 Department of Surgery, University of Otago Christchurch, Christchurch, New Zealand; 2 School of Natural & Computational Sciences, Massey University, Auckland, New Zealand

**Keywords:** CMS classification, colorectal adenocarcinoma, hepatic metastasis, incongruity, molecular subtypes

## Abstract

**Aim:**

This pilot study aimed to evaluate the congruency in consensus molecular subtypes (CMS) of primary colorectal cancer and corresponding hepatic metastasis (HM).

**Materials & methods:**

RNA was extracted from both primary colorectal cancer and HM from ten patients, sequenced to establish gene-expression profiles and classified into CMS. Clinical data were collected retrospectively.

**Results:**

Of the ten patients recruited, nine had primary tumors that were classifiable: seven were CMS2, one was CMS3 and one was CMS4. Five had incongruent classification in the corresponding HM. Three out of the five patients with incongruent classification had received adjuvant chemotherapy prior to hepatic resection.

**Conclusion:**

A change in CMS type between matched primary and metastatic colorectal tumors is common and may be attributable to chemotherapy.

## Lay abstract

Colorectal cancer can be classified into different subtypes and these subtypes can give useful information about the prognosis of disease. Mortality from colorectal cancer often results from tumors spreading to the liver, but little is known about subtypes of the liver metastases. In this preliminary study, we investigated whether colorectal tumors and liver metastases from the same patient had the same subtype profile and whether chemotherapy might cause a change in subtype. This has implications for understanding the effect of chemotherapy on tumors, and whether subtyping metastases gives useful clinical information.

Colorectal adenocarcinoma (CRC) is a complex, heterogeneous disease. Progress has been made to better understand the genomic profiling and classification of CRC [1]. In 2015, the Colorectal Cancer Subtyping Consortium described a novel classification system for CRC [1]. By utilizing genome wide analysis, recurring patterns were identified. CRCs were reclassified based on these recurring patterns into four distinct consensus molecular subtypes (CMS 1, 2, 3 and 4). It is still uncertain whether CMS is preserved when metastasis occurs and how this impacts clinical outcomes. Only a few studies have investigated this [2,3]. In this pilot study, the aim was to assess the congruency of CMS in both primary tumors and their hepatic metastasis (HM) and to lay the foundation for large-scale study.

## Materials & methods

### Patient selection

Ten treatment-naive patients who underwent primary CRC resection and hepatic resection for whom both tissues were available from the Cancer Society Tissue Bank were selected. Three of the patients received chemotherapy following surgery for primary CRC resection, but prior to hepatic resection.

### Nucleic acid extraction, sequencing & classification

Tumor tissue was flash frozen with liquid nitrogen after resection and stored at -80°C. Selected tissue was transferred to RNAlater ICE^®^ (Qiagen NV, Hilden, Germany) and stored at -20°C. RNA was extracted using RNeasy^®^ kit (Qiagen NV) from 20 mg of frozen tumor tissue. Quantification of purified nucleic acid was performed using NanoDrop 2000c spectrophotometer (Thermo Scientific, NC, USA). Sample preparation, including library creation and ribosomal RNA depletion, was carried out using Illumina TruSeq Stranded Total RNA HT reagents (Illumina, CA, USA). RNA sequencing was carried out using the Illumina HiSeq 2500 V4 platform. Raw sequence reads were adapter trimmed using the fastq-mcf program of ea-utils (V1.1.2.779) and quality controlled using SolexaQA++ (v3.1.7.1) with default parameters, as previously described [4]. Transcript expression was quantified using Salmon (v0.11.2) and gene expression in tags per million was calculated using the Tximport package (v1.6.0). Gene expression profiles from each patient were used as input to the publicly available CRC subtype classifier (v1.0.0, https://www.synapse.org/#!Synapse:syn4961785) [1] and classified into four subtypes.

### Clinical data collection

The clinicopathological and 5-year treatment outcome data were collected retrospectively from patient notes. Tumors located proximal to the splenic flexure were documented as 'right-sided’ tumors, tumors located distal to the splenic flexure and proximal to the rectosigmoid junction were documented as ‘left-sided’ tumors and tumors located distal and including rectosigmoid junction were documented as ‘rectal tumors’.

### Data analysis

Statistical analysis using Fisher’s exact test was carried out for categorical data. These were performed using Prism v.8.4.1 (GraphPad). Test results with a p-value of <0.05 were deemed significant.

## Results

### Cohort characteristics

Table 1 shows the clinical characteristics of the ten patients. Nine patients had primary CRCs that were classifiable. Five had synchronous HM and five had metachronous HM. Three patients underwent adjuvant chemotherapy (FOLFOX) prior to hepatic resection.

**Table 1.** Demography and clinical features of patients who had both colonic resection and liver resections.

	Gender	Site	Adjuvant therapy	Primary CMS	Liver CMS
1	Male	Right colon	No	2	2
2	Male	Left colon	Yes	2	4
3	Male	Left colon	No	2	2
4	Male	Left colon	No	3	NC
5	Female	Right colon	Yes	2	4
6	Male	Left colon	No	2	2
7	Female	Rectum	Yes	2	4
8	Male	Right colon	No	4	2
9	Male	Right colon	No	2	2

CMS: Consensus molecular subtype; NC: Not classified.

### CMS classification

Of the ten patients studied, nine had primary tumors that were classifiable using CMS; seven were CMS2 and the remaining two were CMS3 and CMS4, respectively. No CMS1 was identified (Table 1). Of the nine patients who had classifiable primary tumor, incongruity occurred in five. As shown in Table 1, three ‘switched’ from CMS2 to CMS4, one ‘switched’ from CMS3 to unclassifiable and one ‘switched’ from CMS4 to CMS2. Although the association between adjuvant therapy and CMS ‘switching’ was not significant (p = 0.167), three of the five incongruent HM had received adjuvant therapy prior to liver resection. Importantly, the tumors of the three patients who received adjuvant chemotherapy switched from CMS2 to CMS4.

## Discussion

Multiple studies have investigated the genetic profiling of both CRCs and their paired HM [3,5–7]. To date little is known about the congruity of the genetic profiling and how this impacts clinical outcomes and response to treatment. Our pilot study was designed as a feasibility study to investigate the congruency of the molecular profiling between primary CRC and their HM by classifying them according to the CMS and to assess the potential effects of adjuvant chemotherapy.

Two key findings were identified. First, the HM within this cohort were mainly CMS2 subtypes. CMS2 tumors tend to have a higher somatic copy-number alteration counts and are associated with activation of the WNT and MYC signaling pathways [1]. This finding supports those of Sayagues *et al.* who reported upregulation of genes associated with these canonical pathways in colorectal HM [7]. Similarly, Ostrup *et al.* and Pitroda *et al.* reported high prevalence of CMS2 subtypes and the relative absence of CMS1 subtypes in colorectal HM [2,3]. Piskol *et al.* recently published a retrospective analysis of a large cohort of 107 matched primary and metastatic tumors from different sites [8]. Of the 34 patients who had metastases to the liver, 23 liver samples were subtyped as CMS2 (68%). It is postulated that the relatively indolent immune nature of CMS2 tumors allows it to escape the adaptive immune response; thereby, facilitating the ability to metastasize [1,2].

The second key finding is the incongruity in the molecular subtyping of HM which was shown in a substantial proportion of our cohort. The study by Piskol *et al.* found a high level of overall concordance between primary and metastatic tumor samples. However, when examining solely the tumors that metastasised to the liver, 16 out of 34 patients (47%) had incongruous CMS, similar to the rates of incongruency reported here (50%) [8].

More importantly is the strong association with neoadjuvant chemotherapy in three of the five incongruent cases. All three cases had a ‘switch’ to CMS4 subtype. In contrast, four out of six patients who did not receive neoadjuvant therapy had congruent molecular classification. As reported by the Colorectal Cancer Subtyping Consortium, CMS4 tumors tend to have a mesenchymal histology and are associated with activation of epithelial–mesenchymal transition (EMT) genes [1]. Our findings reflect those of a study by Trumpi *et al.* who utilized immunohistochemistry to classify tumors into epithelial-like tumors and mesenchymal-like tumor. Their findings suggested that adjuvant therapy leads to ‘switching’ of subtyping within the HM to a mesenchymal-like subtype [9]. Our study provides further support to the idea of intratumoral heterogeneity; each tumor consisting of a multitude of subclonal cells, each having distinct biological and molecular properties. Selection pressure from neoadjuvant chemotherapy leads to a mesenchymal predominant subclonal population [9], hence a ‘switch’ in CMS. This is also seen in other epithelial malignancies [10]. Evidence suggests that neoadjuvant therapy has led to incongruence in the molecular classification between primary breast cancer and distant metastases and a change in the HER2 status [10,11].

CMS4 tumors have been shown to have a worse prognosis compared with other subtypes [1]. Whether this chemotherapy induced ‘switch’ to CMS4 tumor will lead to a worse clinical outcome has yet to be assessed. There is evidence to suggest that HM with stomal and mesenchymal signature and dysregulation of EMT genes are associated with poor survival outcomes [2]. The benefits resulting from downstaging and improved resectability as a result of neoadjuvant chemotherapy must be weighed up against the potential poor prognosis associated with CMS4 subtype switching.

## Conclusion

This pilot study included only ten patients, making it difficult to draw any strong conclusions. Despite this, an observation of a predominant CMS2 subtype in HM and CMS incongruity associated with neoadjuvant chemotherapy has been demonstrated. Further large-scale studies are needed, to first validate the subtype incongruity associated with neoadjuvant chemotherapy and second, to investigate if this switch in subtype is associated with a poorer clinical outcome.

## Future perspective

This pilot study showed incongruity in CMS between primary CRC and HM. It raises the possibility that neoadjuvant chemotherapy induces switching of CMS in these metastases. The potential impact on survival and prognosis needs to be further evaluated in a large cohort. The advent of digital spatial profiling will facilitate investigation of molecular signatures from different elements in the tumor micro-environment and help resolve the role of chemotherapy in CMS switching.

**Summary points**

- Consensus molecular subtypes (CMS) were assigned to ten treatment-naive primary colorectal tumors and their matched liver metastases, using gene-expression data.
- Five of nine classifiable primary tumors had incongruent subtypes in their liver metastases.
- Three of the incongruent tumors pairs had ‘switched’ from CMS2 to CMS4.
- These same three patients had received adjuvant chemotherapy prior to liver tumor resection.
- These findings suggest a possible impact of adjuvant chemotherapy on the molecular subtype of colorectal cancer and future large-scale studies are warranted.
